# Winter peridermal conductance of apple trees: lammas shoots and spring shoots compared

**DOI:** 10.1007/s00468-012-0826-0

**Published:** 2012-12-14

**Authors:** B. Beikircher, S. Mayr

**Affiliations:** Institute of Botany, University of Innsbruck, Sternwartestraße 15, 6020 Innsbruck, Austria

**Keywords:** Drought, Embolism, Freeze-thaw, Growth, Vulnerability, Tree hydraulics, *Malus*

## Abstract

Lammas shoots are flushes formed by some woody species later in the growing season. Having less time to develop, tissue formation is suggested to be incomplete leading to a higher peridermal water loss during consecutive months. In this study, we analysed morphological and anatomical parameters, peridermal conductance to water vapour and the level of native embolism in mid-winter and late-winter of lammas shoots and normal spring shoots of the apple varieties *Malus domestica* ‘Gala’ and ‘Nicoter’. Lammas shoots showed a significantly higher shoot cross-sectional area due to larger pith and corticular parenchyma areas. In contrast, phloem was significantly thicker in spring shoots. No pronounced differences were observed in xylem and collenchyma thickness or mean hydraulic conduit diameter. The phellem of spring shoots was composed of more suberinised cells compared to lammas shoots, which led to a significantly higher peridermal conductance in the latter. The amount of native embolism in mid-winter did not differ between shoot types, but in late-winter lammas shoots were more embolised than spring shoots. Data show that the restricted vegetation period of lammas shoots affects their development and, in consequence, their transpiration shield. This may also pose a risk for winter desiccation.

## Introduction

Lammas shoots are bursts of shoot growth which, in contrast to spring shoots, occur later in the growing season. Usually, woody plants of the temperate zone start their growth in spring after termination of winter dormancy and when environmental conditions are favourable. Shoots of previous year buds elongate and new buds are formed, out of which shoots develop in the following growing season (Larcher 2003). In some trees, these newly formed buds open already in the current growing season, producing a late-season burst of growth (Pallardy 2008). This phenomenon is mostly known as ‘lammas shoots’, as they often appear around Lammas day on August 1 (Evans 1972; Guédés 1981; Kozlowski and Pallardy 2002; Larcher 2003; Meier 2003). However, other terms like ‘Johannis shoots’ (Kobel 1954; Lyr et al. 1967; Weinreich 2000) or ‘summer flushes’ (Sabatier and Barthélémy 2001) are also used and in some species, even more than two flushes per growing season can occur (Jones 1959; Barthélémy and Caraglio 2007; Cline and Harrington 2007; for a critical revision of terms also see Caraglio and Barthélémy 1997). The occurrence of lammas shoots is most probably endogenously controlled, but also depends strongly on environmental conditions, such as water and nutrient supply during the growing season (Kobel 1954; Jones 1959; Kozlowski 1964; Simak 1970; Kolb and Matyssek 2001; Kozlowski and Pallardy 2002; Codesido and Fernandez-Lopez 2009).

Lammas shoots have been reported for many genera in gymnosperms and angiosperms (Kozlowski 1964; Kozlowski and Pallardy 2002; Pallardy 2008), amongst them also in *Malus domestica* (Kobel 1954; Friedrich and Fischer 2000; Meier 2003). In this species, depending on cultivar, cultivar-stock-combination, tree vigour and cultivation conditions lammas shoots can occur until late in the growing season (Friedrich and Fischer 2000). In commercial apple growing, these shoots are unwanted as they can negatively influence trees: It has been known since long that lammas shoots are susceptible to frost injury (Kobel 1954; Jones 1959; Kozlowski and Pallardy 2002; Pallardy 2008;). This may be related to a failed growth termination which is the first prerequisite for frost hardening (Larcher 2003) and can lead to the shoots’ dieback. Furthermore, apple growers in Northern Italy have observed that trees with numerous lammas shoots sometimes show delayed or even failed leaf flushing in spring. As this occurred mostly on sites with elongated soil frost (e.g. northern exposed or shadowy sites) it has been suggested that winter desiccation may play a role.

Winter desiccation occurs when frozen soils inhibit water uptake and thus evaporative water losses cannot be compensated (Larcher 2003). These water losses can occur not only over stomata but also over the periderm. In deciduous angiosperms, which shed their leaves in autumn, winterly water loss over the periderm can amount to about 16 % of their water content (Friedrich and Fischer 2000). The periderm is the outermost layer of secondary above-ground organs and consists of a cork cambium (phellogen) that produces a multicell outer layer of cork cells (phellem) and an inner layer of cork parenchyma (phelloderm; Lendzian 2006; Barclay 2007; Wittmann and Pfanz 2008). At maturity, phellem cells are dead and their walls are covered with suberin and waxes, thus providing an efficient protection against water loss. Due to the relatively short growing time, lammas shoots fail to develop to maturity (Kobel 1954; Kozlowski 1964) and it can be assumed that their peridermal shield is also not sufficiently developed. This could lead to increased water loss during autumn and winter and, in consequence, force winter desiccation. This drought stress can be amplified when the xylem conduits are impaired by embolism (e.g. Sperry and Sullivan 1992), which is induced by frost drought and freeze-thaw events (Sperry and Sullivan 1992; Sperry et al. 1994; Tyree and Cochard 1996; Mayr et al. 2003, 2007).

To our knowledge there have been no controlled studies dealing with peridermal water loss of lammas shoots and only few with ‘regular’ shoots (e.g. Kozlowski 1943; Geurten 1969; Cernusak and Marshall 2000; Pfanz et al. 2002; Saveyn et al. 2008; Wittmann and Pfanz 2008). The aim of this study was thus to analyse the peridermal conductance to water vapour of apple shoots in winter. We thereby distinguished between (I) normal ‘spring shoots’, which derive from winter buds at the beginning of the growing season and (II) ‘lammas shoots’, which develop from the newly formed terminal bud of the spring shoot later in the season. We also studied the level of native embolism and linked hydraulic data to anatomical and morphological features. Our findings indicate that lammas shoots are less developed and show a higher peridermal conductance, which may pose a risk for winter desiccation and delay embolism repair in spring. Measurements were made on the apple varieties Gala and Nicoter which differ in maturing time. The results of this study are also highly relevant to grower practices with apple orchards in north temperate climates.

## Materials and methods

### Plant material and microclimate

**Fig. 1:**
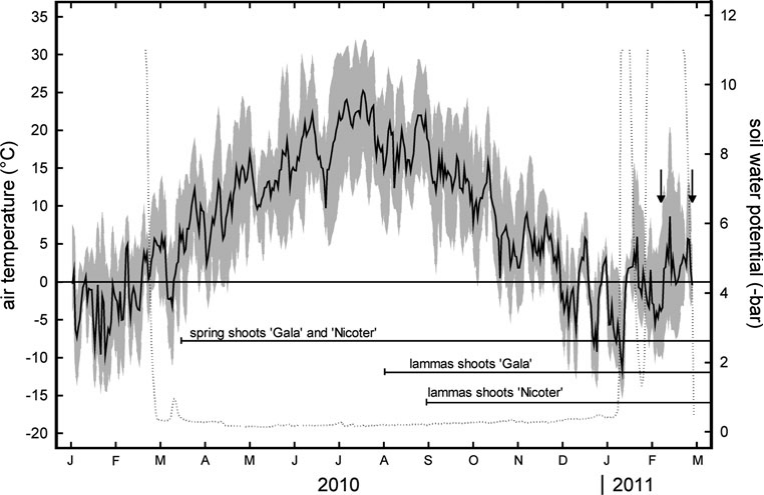
Daily mean (*solid line*), maximum and minimum (*grey area*) of air temperature (°C) and daily mean of soil water potential (*dotted line*) in 15 cm depth at the study site from January 2010 to March 2011. *Horizontal arrows* indicate time of development for spring shoots and lammas shoots of Gala and Nicoter, respectively. *Vertical arrows* show sampling dates. Please be aware that soil water potential values may be biased during freezing and thawing

Measurements were made in winter on leafless spring shoots and lammas shoots of the apple varieties *Malus domestica* ‘Gala’ and *Malus domestica* ‘Nicoter cov’ (fruit trade name Kanzi^®^), growing site by site in an apple orchard in Northern Italy (Latsch, South Tyrol, 600 m a.s.l.; 46°37′N, 10°52′E). Gala is harvested mid of August, while Nicoter requires about 1 month more for fruit ripening. Trees were about 5 (Nicoter) and 9 (Gala) years old, up to 3 metres tall and had a DBH (i.e. diameter at breast height) of 10.5 ± 2 cm. Air temperature in the orchard was registered with an air temperature and humidity sensor (EMS 33) fixed at a height of 250 cm (upper crown) and soil water potential in 15 cm depth with a gypsum block (GB2). Actual values were registered in 1 min intervals and 15 min means were stored with a datalogger (ModuLog 3029; sensors and datalogger of EMS, Brno, Czech Republic). The apple orchard is situated in an inner Alpine dry valley with exceptionally high sun shine duration (315 days), high annual mean temperature (9.6 °C) and low precipitation (481 mm). From November to March air temperatures normally decrease below 0 °C, although even in mid-winter considerable spells of warm weather can occur (see Fig. 1, winter 2011). On northern exposed sites such as in the present study, minimum air temperatures of about −15 °C occur and soils can be frozen for several weeks.

### Morphology and anatomy

**Fig. 2:**
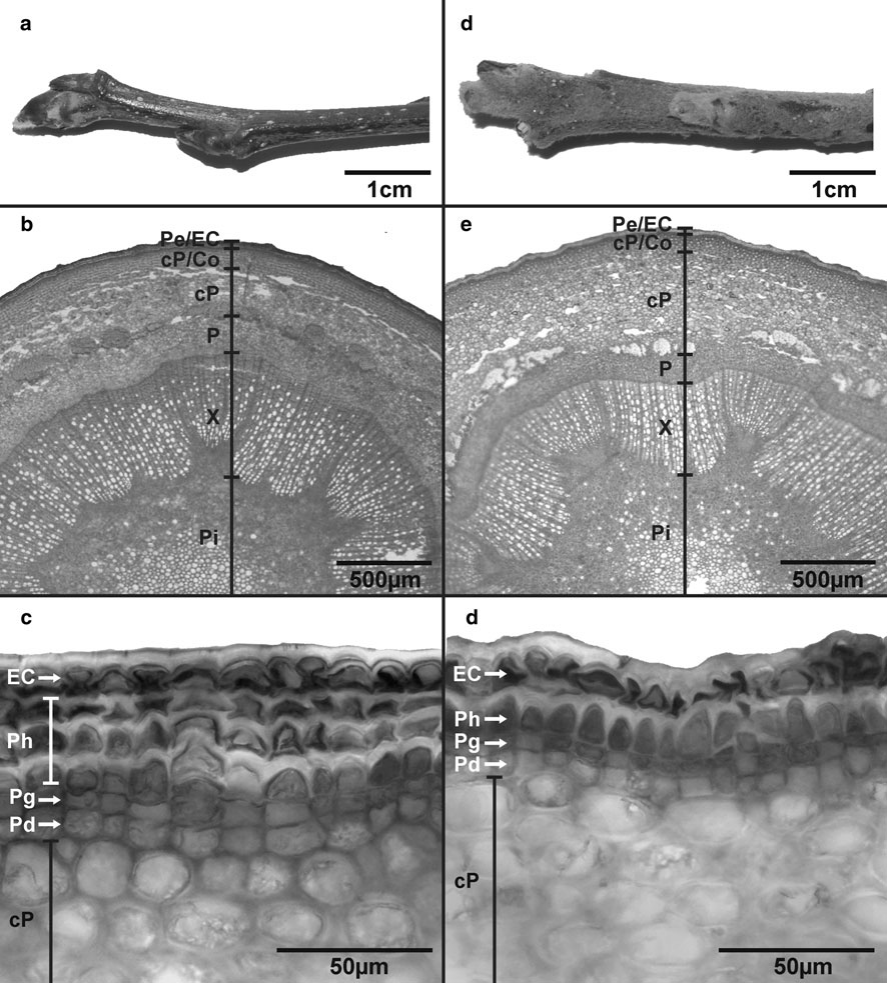
Morphology (**a**, **d**), shoot cross section (**b**, **e**) and detail of the outermost layer (**c**, **f**) of spring shoots (**a**–**c**) and lammas shoots (**d**–**f**) of Nicoter. In the lowermost pictures, suberinised phellem cells and the cuticle appear *light grey* due to staining with sudan-III-glycerol. (*Pe* Periderm, *EC* epidermis and cuticle, *cP* corticular parenchyma, *Co* collenchyma, *P* phloem, *X* xylem, *Pi* pith, *Ph* phellem, *Pg* phellogen, *Pd* phelloderm)

Branches with spring shoots and lammas shoots were harvested on 31 January 2011, recut twice under water (about 3 cm), wrapped in dark plastic bags and transported to the laboratory, where they were recut again (about 1 cm) under water and saturated for 24 h at room temperature. Afterwards, spring shoots and lammas shoots were cut off the branches under water. The first centimetre on the shoots’ basal end was taken for morphological and anatomical measurements. The remaining shoot was then used for conductance measurements (see below). Samples for anatomical analyses were soaked in an ethanol–glycerol–water solution (1:1:1, v/v/v) for 3 weeks before further measurements. Cross sections were cut using a microtome (Sledge Microtome G.S.L. 1, Schenkung Dapples, Zurich, Switzerland) and stained with Etzold’s staining solution (stains lignin red; Etzold 1983) or sudan-III-glycerol (stains suberin, cutin and waxes brightly orange; Wanner 2004). Anatomical parameters were analysed with a light microscope (Olympus BX 41, System Microscope, Olympus Austria, Vienna, Austria) interfaced with a microscope camera (ProgRes^®^ CT3, Jenoptik, Jena, Germany) and image analysis software (ImageJ, 1.37, National Institutes of Health (NIH), Bethesda, USA, public domain). For each cross section stained with Etzold’s staining solution, shoot area was measured as well as radii (in the following referred to as thickness) of pith, xylem, phloem, corticular parenchyma and collenchyma layers. As thickness of different layers is not always even, thickness was measured on five different sites per cross section and averaged. In the case of xylem, these measurements were made at the dents of the pith (see Fig. 2b, e). Neither in spring shoots nor in lammas shoots eccentric growth was observed and thus measurement sites were evenly distributed over the cross section. In a randomly chosen radial section, all individual conduit and corticular parenchyma areas (i.e. lumen, A) were analysed. Respective diameters (d) for conduits were then calculated from A assuming that the conduits had a circular shape (Beikircher and Mayr 2009):

1 $$ d = 2*\surd (A/\pi ) $$

The mean hydraulic conduit diameter (*d*_h_) was calculated from the individual diameters according to Sperry and Hacke 2004):

2 $$ d_{\text{h}} = \frac{{\mathop \sum \nolimits d^{5} }}{{\mathop \sum \nolimits d^{4} }} $$

On samples stained with sudan-III-glycerol, thickness of the layer composed of suberinised phellem cells, epidermis and cuticle was measured as well as number of radial phellem cell rows counted on five different sites per cross section and averaged.

### Peridermal conductance to water vapour (*g*_p_)

Shoots for conductance measurements were de-barked at the basal end (about one cm), recut with a sharp wood carving knife and sealed in a modified Sperry apparatus (see below). The distal part of the shoot with the terminal bud was then cut off under water and the sample was flushed at a pressure of 80 kPa for 45 min to remove possible air bubbles. Wet end parts of the samples were cut off and sample ends were tightly sealed with Parafilm^®^ ‘M’ (Pechiney Plastic Packaging, Menasha, Wisconsin, USA) to prevent water loss over cut ends. Immediately after sealing, turgid weight (TG) of each sample was measured with an analytical balance (Sartorius BP61S, 0.0001 g precision, Sartorius AG, Göttingen, Germany). Samples were then put in a test tube rack for dehydration on the bench and fresh weight (FW) was measured at regular intervals. Air humidity, air temperature and barometric pressure were measured with a thermo-hygrometer (RS Components Handelsgesm.b.H., Gmünd, Austria) and a temperature-compensated pressure sensor (SCX15ANC; Honeywell Sensing and Control, Golden Valley, Minnesota, USA). After 20 days, sample length and diameter were taken and samples were dried at 80 °C for 48 h to obtain the dry weight (DW). From fresh weight (FW), turgid weight (TW) and dry weight (DW), the relative water content (RWC) was calculated as:

3 $$ {\text{RWC}} = ({\text{FW}} - {\text{DW}})/({\text{TW}} - {\text{DW}})*100 $$

For each time interval, evaporation (*E*) was calculated as

4 $$ E = (\Updelta FG/\Updelta t)/(A*m_{{{\text{H}}_{2} {\text{O}}}} ) $$

where Δ*FG* is the difference in fresh weight at a given time interval (Δ*t*), $m_{{{\text{H}}_{2} {\text{O}}}}$ is the molecular mass of water (18.01528 g mol^−1^) and *A* is the transpiring surface area calculated of sample length (*h*) and radius (*r*) assuming a cylindrical shape (Eq. 5)

5 $$ A = 2\pi rh $$

Peridermal conductance (*g*_p_) at a given time was then calculated as

6 $$ g_{\text{p}} = E/(({\text{SVP}} - {\text{VP}})/P)) $$

where SVP (Pa) is the saturated vapour pressure, VP (Pa) is the actual vapour pressure and *P*(Pa) is the barometric pressure (also see Mayr et al. 2010).

### Native embolism

Analyses of native embolism were performed on shoots collected on 31 January 2011 (mid-winter) and 21 February 2011 (late-winter). In contrast to the samples for conductance measurements, these shoots were not saturated. Level of native embolism was analysed by measuring the percentage loss of hydraulic conductivity (PLC) with a modified Sperry apparatus (Sperry et al. 1988; Chiu and Ewers 1993; Mayr et al. 2002). About 3 cm long samples were cut out of the shoot under water, de-barked, re-cut with a sharp wood carving knife and sealed under water in the silicone tubes of the measurement system. Measurement pressure was set to 4 kPa and the flow rate was determined with a PC-connected analytical balance (specification see above) by weight registration every 10 s and linear regression over 200 s. For measurements, distilled, filtered (0.22 μm) and degassed water containing 0.005 % (v/v) ‘Micropur Forte MF 1000F’ (a mixture containing Ag+ and sodium hypochlorite sold for water sterilization and preservation; Katadyn Products Inc., Wallisellen, Switzerland) to prevent microbial growth (Sperry et al. 1988; Beikircher and Mayr 2008) was used. Between measurements of the flow rate, samples were flushed at 80 kPa for 20 min. Flushing was repeated until measurements showed no further increase in conductivity. PLC was calculated as percentage flow rate of the first measurement (*k*_*i*_) in comparison to maximum flow rate (*k*_max_):

7 $$ {\text{PLC}} = 100 - (k_{i} /k_{\hbox{max} } *100) $$

### Number of samples and statistics

Peridermal conductance and anatomical parameters were analysed on eight to ten and the level of native embolism on five shoots per variety and shoot type. Anatomical measurements were made on one cross section per shoot. Mean hydraulic diameter (*d*_h_) was calculated of a total of 633–1,202 conduits and averaged from the means for each single shoot.

Differences between shoot types within a variety were tested with the Student’s *t* test (normal distribution and equal variances) or the Mann–Whitney *U* Test (no normal distribution and/or unequal variances). All tests were made at a probability level of 5 %.

## Results

In both cultivars, bud break in spring 2010 occurred around 15 March, when mean daily air temperature no longer decreased below zero (Fig. 1). In contrast, lammas shoots started growing at the beginning (Gala) or at the end (Nicoter) of August 2010.

Lammas shoots had a significantly higher shoot area due to a higher pith area and a thicker corticular parenchyma layer (Table 1; Fig. 2). In contrast, phloem was significantly thicker in spring shoots. For xylem and collenchyma layers, no pronounced difference in thickness was observed. However, except for corticular parenchyma ratios between thickness of shoots and those of different tissues were relatively constant between spring shoots and lammas shoots (Table 1).

**Table 1:** Shoot area, thickness (i.e. radius) of pith, xylem, phloem, corticular parenchyma and collenchyma layers, area of corticular parenchyma cells, number of suberinised periderm cells along radial sequences and mean hydraulic conduit diameter (*d*_h_) of spring shoots and lammas shoots of Gala and Nicoter

	Gala		Nicoter	
	Spring shoot	Lammas shoot	Spring shoot	Lammas shoot
Shoot area (mm^2^)	13.53 ± 0.43*	17.38 ± 0.64	16.13 ± 1.14*	24.92 ± 1.48
Pith thickness (μm)	734 ± 13* (0.35)	852 ± 25 (0.36)	879 ± 33* (0.37)	1,035 ± 51 (0.38)
Xylem thickness (μm)	609 ± 37 (0.29)	630 ± 48 (0.27)	638 ± 46 (0.27)*	613 ± 78 (0.23)
Phloem thickness (μm)	241 ± 8* (0.12)	186 ± 8 (0.08)	219 ± 11* (0.09)	182 ± 10 (0.07)
Corticular parenchyma thickness (μm)	294 ± 13* (0.14)	422 ± 19 (0.18)	352 ± 30* (0.15)	592 ± 29 (0.22)
Collenchyma thickness (μm)	89.0 ± 3.3	83.2 ± 3.6	97.0 ± 4.5	91.0 ± 5.3
Area corticular parenchyma cells (μm^2^)	878 ± 85	966 ± 82	1,102 ± 71	1,346 ± 96
Number of suberinised periderm cells	3.00 ± 0.12*	2.31 ± 0.16	2.94 ± 0.13*	2.07 ± 0.07
*d*_h_ (μm)	23.18 ± 0.0003*	24.70 ± 0.0003	24.31 ± 0.0003*	23.62 ± 0.0003

Values in parenthesis give the ratio to shoot thickness. Asterisks indicate significant differences between shoot types within each apple variety (*P* < 0.05). Mean and SE

**Fig. 3:**
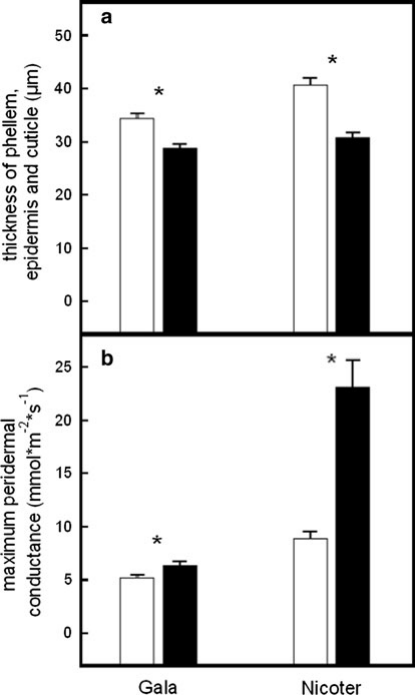
Thickness of the layer consisting of suberinised phellem cells, epidermis and cuticle (**a**) and maximum peridermal conductance of spring shoots (*open bars*) and lammas shoots (*solid bars*; **b**) of Gala and Nicoter. *Asterisks* indicate significant differences between shoot types within each apple variety (*P* = 0.05). Mean and SE

In Gala, mean hydraulic conduit diameter (*d*_h_) was significantly higher in lammas shoots compared to spring shoots, while in Nicoter spring shoots showed higher *d*_h_ (Table 1). The outermost layer, i.e. phellem, epidermis and cuticle was significantly thicker in spring shoots than in lammas shoots due to a higher number of suberinised phellem cells (Figs. 2, 3a; Table 1).

**Fig. 4:**
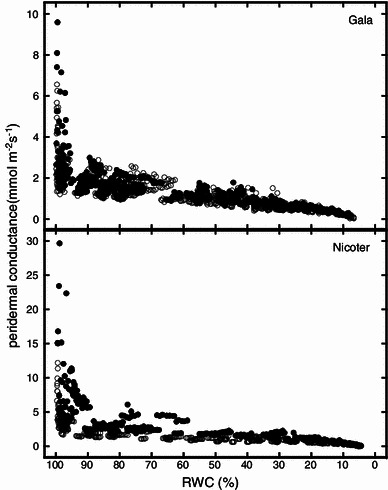
Peridermal conductance versus relative water content (RWC) of spring shoots (*open circles*) and lammas shoots (*closed circles*) of Gala and Nicoter

In both cultivars, lammas shoots showed significantly higher maximum peridermal conductance (*g*_p_). In Nicoter, lammas shoot values were more than twofold that of spring shoots (Fig. 3b). With decreasing relative water content (RWC) a rapid decrease in *g*_p_ was observed in both cultivars until ca. 98 % RWC. Down to an RWC of about 55 %, lammas shoots of Nicoter showed an overall higher *g*_p_ during desiccation than spring shoots, while no obvious differences were found for Gala (Fig. 4).

**Fig. 5:**
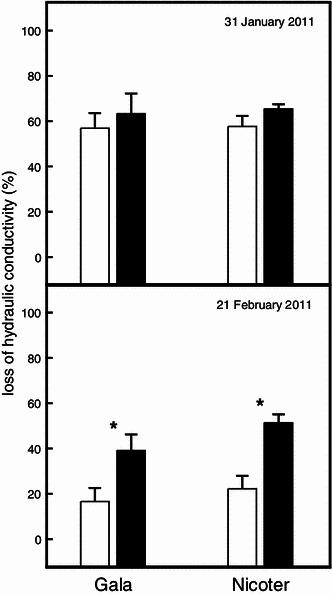
Percentage loss of hydraulic conductivity on-site (i.e. native embolism) on 31 January 2011 and on 21 February 2011 of spring shoots (*open bars*) and lammas shoots (*solid bars*) of Gala and Nicoter. *Asterisks* indicate significant differences between shoot types within each apple variety (*P* < 0.05). Mean and SE

In both apple varieties, native embolism in mid-winter was only slightly higher in lammas shoots, while in late-winter level of native embolism was more than twice as high in lammas shoots (Fig. 5).

## Discussion

In North Italian apple orchards, lammas shoots are known to occur on vigorous trees when rainy periods in spring and early summer are followed by a warm and sunny mid-summer. The onset of lammas shoot formation is thereby consistent neither between years nor between varieties. In our study, lammas shoots of Gala sprouted about 18 weeks after spring shouts and 3 weeks earlier than those of the nearby growing Nicoter trees (Fig. 1).

Up to the following growing season, lammas shoots on apple trees can be clearly morphologically distinguished from spring shoots: First of all, they are overall thicker and softer which is mainly due to a significantly higher pith area and a thicker layer of corticular parenchyma (Table 1; Fig. 2). Cells of the latter are also slightly larger compared to spring shoots although differences were not significant. Second, lammas shoots are remarkably hairy (Fig. 2) and leaves are smaller, of a light green colour and often remain connected to the shoot until late in winter. This incomplete leaf abscission could negatively influence tree hydraulics in autumn by uncontrolled water loss over death leaves still connected to the shoot xylem. However, in winter conduits in the petiole are supposed to be embolised and thus water loss over these leaves is negligible. In our study, only leafless shoots were analysed in winter months. Anatomical analyses revealed that in both shoot types, epidermis and cuticle were still intact, but in spring shoots the subjacent phellem was composed of more suberinised cells than in lammas shoots and, in consequence, formed a thicker peridermal layer (Figs. 2, 3a). In both varieties, the less developed periderm in lammas shoots resulted in a significantly higher maximum peridermal conductance (*g*_p_; Fig. 3). These findings are also in accordance with Wittmann and Pfanz 2008), who found stem transpiration in current-year shoots of various angiosperms to be strongly correlated with periderm thickness. Also for older stem sections, phellem thickness was found to be well correlated with water permeance (Groh et al. 2002; Lendzian 2006).

There are only few studies dealing with water exchange over woody stems and moreover, most findings of those studies are not directly comparable to our results due to different approaches (e.g. measurements on isolated phellem or periderm by Schönherr and Ziegler 1980; Groh et al. 2002; Lendzian 2006) or non-convertible units (e.g. water exchange rates in grams per week by Kozlowski 1943). There are some studies reporting values for peridermal transpiration of temperate trees, giving a range from about 0.015 to 0.055 mmol m^−2^ s^−1^ (Geurten 1969; Larcher 2003; Saveyn et al. 2008), but to our knowledge Cernusak and Marshall 2000), Wittmann et al. 2006) and Wittmann and Pfanz 2008) are the only studies giving peridermal conductance values. According to Wittmann and Pfanz 2008), *g*_p_ of current-year shoots ranges from 5.01 to 27.3 mmol m^−2^ s^−1^ in five different temperate angiosperm species. Values given for *Prunus avium* (5.01 mmol m^−2^ s^−1^), like *Malus* a Rosaceae, are similar to maximum *g*_p_ measured on spring shoots of Gala and Nicoter (Fig. 3). Down to an RWC of about 98 % *g*_p_ decreased rapidly, especially in lammas shoots (Fig. 4). During further desiccation no obvious differences were found between spring and lammas shoots of Gala, while in Nicoter lammas shoots showed higher *g*_p_ down to an RWC of about 55 %. Altogether, these anatomical and conductance measurements revealed that lammas shoots were more prone to peridermal water loss than spring shoots, but differences between shoot types were more pronounced in Nicoter, where lammas shoots had 3 weeks less time to develop than in Gala.

The shorter development time may not only influence the periderm but also other tissues, such as the phloem and xylem. In both cultivars, phloem was significantly thinner in lammas shoots while for the xylem no pronounced differences in thickness were observed (Table 1). This may be related to different dynamics in tissue formation. According to Evert 1963), phloem differentiation in *Malus* normally precedes xylem differentiation by approximately 1.5 months while cessation occurs almost simultaneously. It can be assumed that these dynamics are altered in lammas shoots due to the overall shorter development time. Onset and cessation of tissue formation may occur more or less contemporaneously resulting in less developed phloem. The shorter development time can also impact tissue maturation. Dietrichson 1964) for instance reports that delayed latewood formation in conifers can lead to incomplete lignification and thus larger frequency of snow breaks. Several studies have shown that, depending on tree species, complete xylogenesis requires about 77–160 days (e.g. Rossi et al. 2007; Deslauriers et al. 2009; Lupi et al. 2010; Romagnoli et al. 2011). It is also known that latewood cells require more time to complete formation because cell wall thickening phase takes longer (Deslauriers et al. 2003). As spring shoots of studied apple varieties had about 5 months more time to develop than lammas shoots, incomplete lignification in the latter is probable. Indeed, several authors have stated that lammas shoots are only imperfectly lignified and thus also more susceptible to frost damage (Kobel 1954; Jones 1959; Kozlowski and Pallardy 2002; Pallardy 2008). In contrast, our analysis on native embolism did not indicate any influence on the xylem’s hydraulic safety. Level of native embolism in mid-winter was overall high (about 60 %), but no pronounced difference between spring shoots and lammas shoots was observed (Fig. 5a). Accordingly, conduit diameters, which determine the resistance to freeze-thaw induced embolism (Sperry and Sullivan 1992; Davis et al. 1999; Pittermann and Sperry 2003) were similar (Table 1). Unfortunately, water potential cannot be determined on leafless shoots during winter due to the small portion of living tissues. A high amount of embolised conduits in winter is quite common in apple (Beikircher et al., unpublished), but this does not necessarily affect tree hydraulics when conduits are refilled in spring (e.g. Sperry and Sullivan 1992; Sperry et al. 1994; Tyree and Cochard 1996; Cavender-Bares and Holbrook 2001; Cochard et al. 2001; Vogt 2001). Our study reveals that lammas shoots were obviously not able to refill like normal spring shoots: The second sampling for native embolism measurements occurred about 1 month before bud break and 3 weeks after the first one, when air temperatures increased to positive values and soil water potential increased to −0.5 bar (Fig. 1). Spring shoots of both apple varieties showed an about 40 % reduction in loss of conductivity, while in lammas shoots PLC remained high (Fig. 5). A possible reason for insufficient refilling may be unfavourable water potentials in lammas shoots caused by their high peridermal water loss. Incompletely developed tissues required for refilling such as the phloem (see Salleo 2004; Nardini et al. 2011) could also be a cause.

Lammas shoots can have positive effects on trees as they increase the leaf area and thus photosynthetic capacity, and significantly add to the height growth in some tree species (Kozlowski 1964; Cline and Harrington 2007; Codesido and Fernandez-Lopez 2009). After defoliation due to herbivory or late spring frosts the ability for multiple flushing is of great advantage (Jones 1959; Cline and Harrington 2007). In contrast, lammas shoots have been found to be more susceptible to pathogens and frost damage in several species (Jones 1959; Kozlowski 1964; Nechwatal et al. 2011). Our study demonstrates that lammas shoots can also have negative impacts on tree water relation as lammas shoots of apple trees show a high peridermal conductance due to an imperfect development of tissues. This may affect hydraulics of the whole tree in late-winter by winter desiccation and in early spring by delayed refilling. It can be assumed that removing of lammas shoots in autumn could prevent negative impacts on tree water relations. However, besides the number of lammas shoots per tree and time of formation, the impact of lammas shoots on tree hydraulics may also depend on factors such as water supply in autumn and spring, water storage capacity, well-timed and successful leaf shedding in autumn and climatic conditions.

## References

[CR1] Barclay G, Roberts K (2007). Plant anatomy. Handbook of plant science.

[CR2] Barthélémy D, Caraglio Y (2007). Plant architecture: a dynamic, multilevel and comprehensive approach to plant form, structure and ontogeny. Ann Bot.

[CR3] Beikircher B, Mayr S (2008). The hydraulic architecture of Juniperus communis L. ssp communis: shrubs and trees compared. Plant Cell Environ.

[CR4] Beikircher B, Mayr S (2009). Intraspecific differences in drought tolerance and acclimation in hydraulics of Ligustrum vulgare and Viburnum lantana. Tree Physiol.

[CR5] Caraglio Y, Barthélémy D, Bouchon J, de Reffye P, Barthélémy D (1997). Revue critique des termes relatifs à la croissance et à la ramification des tiges des végétaux vasculaires. Modélisation et simulation de l′architecture des végétaux.

[CR6] Cavender-Bares J, Holbrook NM (2001). Hydraulic properties and freezing-induced cavitation in sympatric evergreen and deciduous oaks with contrasting habitats. Plant Cell Environ.

[CR7] Cernusak LA, Marshall JD (2000). Photosynthetic refixation in branches of western white pine. Funct Ecol.

[CR8] Chiu ST, Ewers FW (1993). The effect of segment length on conductance measurements in *Lonicera fragrantissima*. J Exp Bot.

[CR9] Cline M, Harrington C (2007). Apical dominance and apical control in multiple flushing of temperate woody species. Can J For Res.

[CR10] Cochard H, Lemoine D, Ameglio T, Granier A (2001). Mechanism of xylem recovery from winter embolism in *Fagus sylvatica*. Tree Physiol.

[CR11] Codesido V, Fernandez-Lopez J (2009). Genetic variation in seasonal growth patterns in radiata pine in Galicia (Northern Spain). For Ecol Manage.

[CR12] Davis SD, Sperry JS, Hacke UG (1999). The relationship between xylem conduit diameter and cavitation caused by freezing. Am J Bot.

[CR13] Deslauriers A, Morin H, Begin Y (2003). Cellular phenology of annual ring formation of *Abies balsamea* in the Quebec boreal forest (Canada). Can J For Res.

[CR14] Deslauriers A, Giovannelli A, Rossi S, Castro G, Fragnelli G, Traversi L (2009). Intra-annual cambial activity and carbon availability in stem of poplar. Tree Physiol.

[CR15] Dietrichson J (1964). The selection problem and growth rhythm. Silvae Genet.

[CR16] Etzold H (1983) Eine kontrastreiche, simultane Mehrfachfärbung für pflanzenanatomische Präparate: Fuchsin-Safranin-Astrablau. Mikrokosmos, 213–219

[CR17] Evans C (1972) The quantitative analysis of plant growth. In: Studies in ecology. Blackwell Scientific Publications, London, p 733

[CR18] Evert RF (1963). The cambium and seasonal development of the phloem in *Pyrus malus*. Am J Bot.

[CR19] Friedrich G, Fischer M (2000). Physiologische Grundlagen des Obstbaues.

[CR20] Geurten I (1969). Untersuchungen über den Gaswechsel von Baumrinden. Forstw Cbl.

[CR21] Groh B, Hubner C, Lendzian KJ (2002). Water and oxygen permeance of phellems isolated from trees: the role of waxes and lenticels. Planta.

[CR22] Guédés M (1981). Corrections and additions to the book ‘Morphology of seed-plants’. Phyton.

[CR23] Jones EW (1959). Biologica flora of the British isles. Quercus L. J Ecol.

[CR24] Kobel F (1954). Lehrbuch des Obstbaus auf physiologischen Grundlagen.

[CR25] Kolb TE, Matyssek R (2001). Limitations and perspectives about scaling ozone impacts in trees. Environ Pollut.

[CR26] Kozlowski TT (1943). Transpiration rates of some forest tree species during the dormant season. Plant Physiol.

[CR27] Kozlowski TT (1964). Shoot growth in woody plants. Bot Rev.

[CR28] Kozlowski TT, Pallardy SG (2002). Acclimation and adaptive responses of woody plants to environmental stresses. Bot Rev.

[CR29] Larcher W (2003). Physiological plant ecology.

[CR30] Lendzian KJ (2006). Survival strategies of plants during secondary growth: barrier properties of phellems and lenticels towards water, oxygen, and carbon dioxide. J Exp Bot.

[CR31] Lupi C, Morin H, Deslauriers A, Rossi S (2010). Xylem phenology and wood production: resolving the chicken-or-egg dilemma. Plant Cell Environ.

[CR32] Lyr H, Polster H, Fiedler HJ (1967). Gehölzphysiologie.

[CR33] Mayr S, Wolfschwenger M, Bauer H (2002). Winter-drought induced embolism in Norway spruce (*Picea abies*) at the alpine timberline. Physiol Plant.

[CR34] Mayr S, Gruber A, Bauer H (2003). Repeated freeze-thaw cycles induce embolism in drought stressed conifers (Norway spruce, Stone pine). Planta.

[CR35] Mayr S, Cochard H, Ameglio T, Kikuta SB (2007). Embolism formation during freezing in the wood of *Picea abies*. Plant Physiol.

[CR36] Mayr S, Schwienbacher F, Beikircher B, Dämon B (2010). Damage in needle tissues after infection with *Chrysomyxa rhododendri* increases cuticular conductance of *Picea abies* in winter. Protoplasma.

[CR37] Meier U, Schartz MD (2003). Phenological growth stages. Phenology: an integrative environmental science.

[CR38] Nardini A, Lo Gullo MA, Salleo S (2011). Refilling embolized xylem conduits: Is it a matter of phloem unloading?. Plant Sci.

[CR39] Nechwatal J, Hahn J, Schönborn A, Schmitz G (2011). A twig blight of understorey European beech (*Fagus sylvatica*) caused by soilborne *Phytophora* spp. For Path.

[CR40] Pallardy SG (2008). Physiology of woody plants.

[CR41] Pfanz H, Aschan G, Langenfeld-Heyser R, Wittmann C, Loose M (2002). Ecology and ecophysiology of tree stems: corticular and wood photosynthesis. Naturwissenschaften.

[CR42] Pittermann J, Sperry JS (2003). Tracheid diameter is the key trait determining the extent of freezing-induced embolism in conifers. Tree Physiol.

[CR43] Romagnoli M, Cherubini M, Prislan P, Gricar J, Spina S, Cufar K (2011). Main phases of wood formation in chestnut (*Castanea sativa*) in central Italy—comparison of seasons 2008 and 2009. Drv Ind.

[CR44] Rossi S, Deslauriers A, Anfodillo T, Carraro V (2007). Evidence of threshold temperatures for xylogenesis in conifers at high altitudes. Oecologia.

[CR45] Sabatier S, Barthélémy D (2001). Bud structure in relation to shoot morphology and position on the vegetative annual shoots of *Juglans regia* L. (Juglandaceae). Ann Bot.

[CR46] Salleo S, Lo Gullo MA, Trifilo P, Nardini A (2004) New evidence for a role of vessel-associated cells and phloem in the rapid refilling of cavitated stems of *Laurus nobilis* L. Plant Cell Environ 27:1065–1076

[CR47] Saveyn A, Steppe K, Lemeur R (2008). Report on non-temperature related variations in CO_2_ efflux rates from young tree stems in the dormant season. Trees.

[CR48] Schönherr J, Ziegler H (1980). Water permeability of *Betula* periderm. Planta.

[CR49] Simak M (1970). Photo- and thermoperiodic responses of different larch provenances (*Larix deciuda* Mill.). Studia Forestalia Suecica.

[CR50] Sperry JS, Hacke UG (2004). Analysis of circular bordered pit function-I. Angiosperm vessels with homogenous pit membranes. Am J Bot.

[CR51] Sperry JS, Sullivan JEM (1992). Xylem embolism in response to freeze-thaw cycles and water stress in ring-porous, diffuse-porous and conifer species. Plant Physiol.

[CR52] Sperry JS, Donnelly JR, Tyree MT (1988). A method for measuring hydraulic conductivity and embolism in xylem. Plant Cell Environ.

[CR53] Sperry JS, Nichols KL, Sullivan JEM, Eastlack SE (1994). Xylem embolism in ring-porous, diffuse-porous, and coniferous trees of northern Utah and interior Alaska. Ecology.

[CR54] Tyree MT, Cochard H (1996). Summer and winter embolism in oak: impact on water relations. Ann Sci For.

[CR55] Vogt UK (2001). Hydraulic vulnerability, vessel refilling, and seasonal courses of stem water potential of *Sorbus aucuparia* L. and *Sambucus nigra* L. J Exp Bot.

[CR56] Wanner G (2004). Mikroskopisch-Botanisches Praktikum.

[CR57] Weinreich A (2000) Qualitätsentwicklung junger Eichen in Bestandeslücken. Dissertation, Albert-Ludwigs-Universität, Freiburg im Breisgau

[CR58] Wittmann C, Pfanz H (2008). Antitranspirant functions of stem periderms and their influence on corticular photosynthesis under drought stress. Trees Struct Funct.

[CR59] Wittmann C, Pfanz H, Loreto F, Centritto M, Pietrini F, Alessio G (2006). Stem CO_2_ release under illumination: corticular photosynthesis, photorespiration or inhibition of mitochondrial respiration?. Plant Cell Environ.

